# Enhancement of Thermal Boundary Conductance of Metal–Polymer System

**DOI:** 10.3390/nano10040670

**Published:** 2020-04-02

**Authors:** Susanne Sandell, Jeremie Maire, Emigdio Chávez-Ángel, Clivia M. Sotomayor Torres, Helge Kristiansen, Zhiliang Zhang, Jianying He

**Affiliations:** 1 NTNU Nanomechanical Lab, Department of Structural Engineering, Norwegian University of Science and Technology (NTNU), 7491 Trondheim, Norway; 2 Catalan Institute of Nanoscience and Nanotechnology (ICN2), (ICN-CSIC) Barcelona, Campus UAB, E08193 Bellaterra, Spain; 3 ICREA—Institució Catalana de Recerca i Estudis Avançats, 08010 Barcelona, Spain

**Keywords:** enhancement of thermal boundary conductance, thermal conductivity of polymer thin films, organic electronics, thermal characterization of polymer, adhesion layer

## Abstract

In organic electronics, thermal management is a challenge, as most organic materials conduct heat poorly. As these devices become smaller, thermal transport is increasingly limited by organic–inorganic interfaces, for example that between a metal and a polymer. However, the mechanisms of heat transport at these interfaces are not well understood. In this work, we compare three types of metal–polymer interfaces. Polymethyl methacrylate (PMMA) films of different thicknesses (1–15 nm) were spin-coated on silicon substrates and covered with an 80 nm gold film either directly, or over an interface layer of 2 nm of an adhesion promoting metal—either titanium or nickel. We use the frequency-domain thermoreflectance (FDTR) technique to measure the effective thermal conductivity of the polymer film and then extract the metal–polymer thermal boundary conductance (TBC) with a thermal resistance circuit model. We found that the titanium layer increased the TBC by a factor of 2, from 59 × 10^6^ W·m^−2^·K^−1^ to 115 × 10^6^ W·m^−2^·K^−1^, while the nickel layer increased TBC to 139 × 10^6^ W·m^−2^·K^−1^. These results shed light on possible strategies to improve heat transport in organic electronic systems.

## 1. Introduction

Today, electronics are embedded in our lives through the use of smartphones, computers and the internet, and this will only continue through the staggering development in artificial intelligence, wearable electronics and the Internet of Things. Electronics constantly evolves towards miniaturization, which puts increasing demands on performance, stability and reliability. To create electronics with high performances and reliability as well as long lifetimes, thermal management is paramount [1]. For the development of flexible and transparent electronics, polymers constitute an integral part of circuits. Among its applications are electronic paper, flexible solar cells, electronic skin and wearable electronics [2,3]. Organic electronics also has the advantage of reducing dependence on silicon and rare earth materials. In organic electronics, thermal management is especially challenging because polymers conduct heat poorly and due to the large number of interfaces between thermally dissimilar materials such as metals and polymers. Phonons are the main conductors of heat in polymers [4], unlike metals, where free electrons dominate heat transfer [5]. Due to this, a thermal resistance arises at the interface [6]. Metal–polymer interfaces will only increase in number as organic electronics develops further and the thermal boundary resistances will contribute significantly to the total thermal resistance, leading to ever-growing issues for thermal management of these devices.

**Figure 1:**
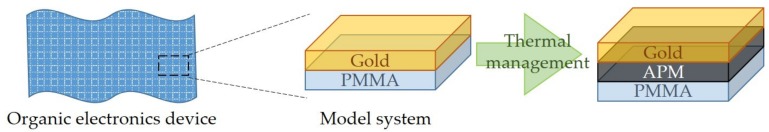
In organic electronics devices, there is an abundance of organic–inorganic interfaces such as the metal–polymer interface. A gold–polymethyl methacrylate (gold–PMMA) interface was chosen as the model system, and an adhesion promoting metal (APM) film was chosen as a way of improving heat transport in the system.

In this sense, it is of the utmost importance to explore novel ways of manufacturing the metal–polymer interfaces while maximizing their thermal boundary conductance (TBC). However, research about thermal management of metal–polymer interface is lacking. In recent years there have been several studies regarding thermal management of organic–inorganic interfaces. The studies show that chemical functionalization [7], HF etching [8], self-assembled monolayers [9] and adhesion promoting layers [10] can all improve the TBC. Among these methods, adding a Ti or Ni nanofilm as an adhesion-promoting layer is an easy and well-known way to increase bond strength between Au and polymers [11]. We have therefore chosen a model system of gold film on polymethyl methacrylate (PMMA), as seen in Figure 1, as a way to study the TBC.

It is not trivial to directly probe the TBC between a polymer and an inorganic material. In the model system, the PMMA layer must be thin enough that the measured conductivity $\kappa_{eff}$ is sensitive to the TBC, instead of being dominated by the bulk contribution. Although the dominant modes of phonon transport in polymers are not well understood, a phonon mean free path value of 0.7 nm has been reported [12]. This means that size effects stemming from reducing the size of the polymer system occur only when the characteristic size approaches this length scale. The reason for the size effect is phonon boundary scattering, which becomes the dominant phonon scattering mechanism when the system size approaches zero [13]. However, since the PMMA layer in this system consists of randomly oriented polymer chains, the phonon mean free path is not the most accurate way of estimating the critical PMMA layer size [14]. Another way of looking at it is to consider the Kapitza length ($\ell_{k}$) in polymers. The low thermal conductivity of polymers, such as 0.20 W·m^−1^·K^−1^ for PMMA, results in a very small $\ell_{k}$

(1) ℓk=κG

which is the characteristic thickness of a film of bulk thermal conductivity κ that gives the same thermal resistance as an interface of TBC G. Assuming κ = 0.20 Wm^−1^K^−1^ and G ≈ 10^8^ W·m^−2^·K^−1^ for PMMA, $\ell_{k}$ = 2 nm [8]. This means that if we want to probe G with appreciable certainty, the thickness of the PMMA layer in the model should be in the range of a few nanometers. For such thin PMMA films, a noncontact characterization method is preferred to avoid damaging the film. We chose frequency-domain thermoreflectance (FDTR) as the thermal characterization technique, since it is an all-optical method suited to measuring G at these length scales.

In this work, we show that the TBC of an Au–PMMA interface can be enhanced by a factor of two by adding a Ti or Ni nanofilm at the interface. We fabricated three series of Si wafer samples with PMMA thickness varying from 1 to 15 nm and deposited an 80 nm Au film on top. On the first series, the Au layer was deposited directly on the PMMA (Au/PMMA/Si). On the second and third sample series, a 2 nm thick layer of Ti or Ni, respectively, was deposited on the PMMA prior to the Au layer deposition (Au/Ti/PMMA/Si and Au/Ni/PMMA/Si, respectively). We measured the effective thermal conductivity of the PMMA films using FDTR and plotted as a function of film thickness. By fitting the results to a thermal resistance circuit, we found the TBC and compared it for the three-sample series. For simplicity the three studied systems, (i) Au/PMMA/Si, (ii) Au/Ti/PMMA/Si and (iii) Au/Ni/PMMA/Si, will be abbreviated as APS, ATPS and ANPS, respectively.

## 2. Materials and Methods

We fabricated samples on 1 × 1 cm^2^ silicon wafer chips cut from the same Si wafer. The chips were cleaned by ultrasonication for 5 min in acetone and isopropanol (both analytical grade, EMSURE, Merck KGaA, Germany), respectively, then rinsed in deionized water and dried in N_2_ stream. A second cleaning step was done by immersing the samples in a piranha etch solution (5:1 H_2_SO_4_/H_2_O_2_) for 20 min followed by the same rinse procedure. We purchased the PMMA powder of average molecular weight of 15,000 Da from Sigma Aldrich (Merck KGaA, Darmstadt, Germany). We dissolved the PMMA in anisol (analytical grade, MicroChem Corp (Westborough, MA, USA)) using magnetic stirring for more than 12 h in a closed container to avoid anisol evaporation. PMMA was spin-coated (Laurell Technologies, North Wales, PA, USA) onto the Si chips at 4000 rpm and cured at 50 °C for 4 h in a vacuum oven (Binder, Tuttlingen, Germany). We varied the thickness of the PMMA layer by changing the weight percentage of PMMA powder in solvent. We measured the PMMA layer thicknesses by variable angle spectroscopic ellipsometry (VASE) (RC2, J.A. Woollam Co., Lincoln, NE, USA) in the wavelength range 210–1690 nm with data collected at angles 55–70°. We deposited the metal films at a rate of 5 Å/s using electron-beam evaporation (AJA International, Inc., Scituate, MA, USA) and measured their thicknesses using atomic force microscopy (Dimension Icon from Bruker, Billerica, MA, USA).

We measured the effective thermal conductivity $\kappa_{eff}$ of the PMMA film using a homemade frequency-domain thermoreflectance (FDTR). FDTR is a pump-probe optothermal technique suited for probing thermal transport at the nanometer scale [15] which is widely used to measure the thermal properties of thin films [16,17,18] and interfaces [19,20,21]. A pump laser periodically heats a metal transducer film and a continuous wave laser measures its reflectivity. The reflectivity change of the transducer is directly linked to its increase in temperature and can be written as *ΔR/R = C_tr_ ΔT*, with *C_tr_* being the thermoreflectance coefficient. This coefficient directly depends on the choice of the transducer/probe laser couple. Then, the signal is detected by a lock-in amplifier that records the amplitude and phase of the probe signal at the modulation frequency of the pump laser. The pump laser was a Cobolt 06-MLD 405 nm (Hübner Group Company, Kassel, Germany) modulated by a HF2 lock-in amplifier (Zürich Instruments, Zürich, Switzerland) from 10 kHz to 10 MHz. The probe laser was a MSL-FN 532 nm (CNI Industries, Changchun, China). For a probe laser wavelength of 532 nm, gold gives the strongest thermoreflectance signal, hence an 80 nm thick gold transducer film was deposited. The reference beam signal and reflected probe beam signal were recorded using identical PDA10A photodetectors (ThorLabs, Newton, NJ, USA) with matched optical path length [15], and we measured the phase difference between the signals using the same lock-in amplifier. We measured the pump and probe laser spot radii using the knife’s edge method. We used an approach similar to Yang et al. in [18], scanning the laser beam across a quartz glass slide with an 80 nm Au layer patterned with a transparent window. We measured the intensity of the reflected light while the slide was translated along the axis perpendicular to the incident laser beam by a servo motor actuator with step size 20 nm. The beam intensity as a function of translation distance was fitted to an error function curve [22] and the 1/e radius of this curve was taken as the laser spot radius. The appropriate laser intensities were determined so that the steady-state temperature rise in the sample would not exceed 3 K [23], which ensured that the measured thermal conductivity could be assumed to be constant in the actual temperature range. A data acquisition program written in MATLAB communicated with instruments. The signal was recorded for 40 modulation frequencies in the 10 kHz–10 MHz range as the frequency directly determines the thermal penetration depth. We then fit the data to a thermal model derived by Cahill [24], which is based on a three-dimensional solution of Fourier’s law for heat conduction through a multilayered structure. The effective thermal conductivity of the PMMA layer, $\kappa_{eff}$, was the only free parameter in the fit. Thermal and dimensional properties that were used in the model are given in Table 1. Thermal measurements were done in three different places close to the center of each sample, since this is where the PMMA thickness was known most accurately.

**Figure 2:**
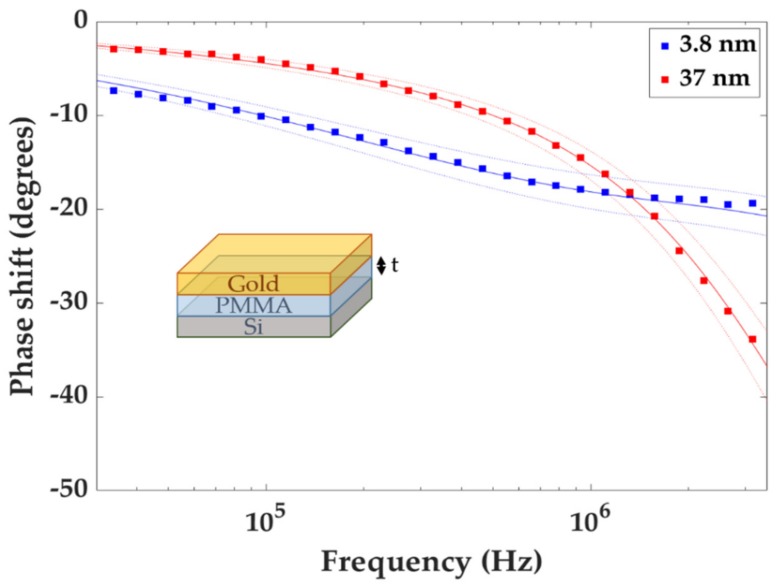
Phase shift is plot as a function of modulation frequency of the pump laser for Au/PMMA/Si (APS) samples with PMMA layer thicknesses 3.8 nm and 37 nm, respectively. The experimental data is then fitted to the thermal model, and the best fit is given as the solid curves using $\kappa_{eff}$ as the fit parameter. In this particular case, for the 3.8 nm PMMA, $\kappa_{eff}$ = 0.1160 W·m^−1^·K^−1^ with 1.64% fit error, and the 37 nm PMMA the $\kappa_{eff}$ = 0.1890 W·m^−1^·K^−1^ with 0.20% fit error. The dotted curves above and below the solid curve correspond to best fit for the $\kappa_{eff}$ ± 10% error.

**Figure 3:**
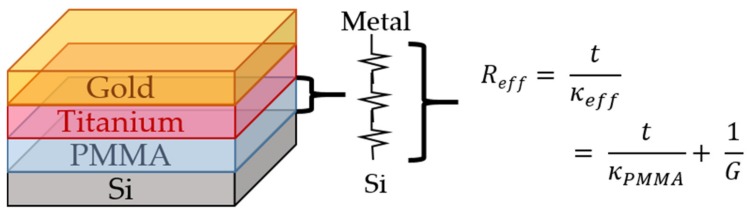
Schematic of a typical sample. The thermal resistance circuit gives a total thermal resistance $R_{eff}$ of the PMMA layer, which is a sum of the interfacial (G *_1,2_*) and bulk ($\kappa_{PMMA})$ contributions. The Si substrate has a native oxide layer which has been omitted in the figure for clarity.

Figure 2 shows an example of typical phase shift vs. frequency data for an APS sample. We then used the thermal model to do a single parameter fit to determine $\kappa_{eff}$ for the PMMA layer in each sample. The best fit curve is also shown in Figure 2, together with the dashed curves above and below it, which is a representation of the best fit curve for $\kappa_{eff}$ ± 10%. Using a thermal resistance circuit illustrated in Figure 3, G is found:

(2) $$\kappa_{e_{ff}}\left(t\right)=t\times {\left(\frac{t}{\kappa_{PMMA}}+\frac{1}{G}\right)}^{-1}$$

where $\kappa_{e_{ff}}\left(t\right)$ is the measured thermal conductivity of the PMMA film of thickness t, $\kappa_{PMMA}$ is the bulk thermal conductivity of PMMA and the G is a sum of the two TBCs interfacing the PMMA layer, $G_{Metal-PMMA}$ and $G_{PMMA-Si}$. Equation (2) can be fit to the measured data $\kappa_{e_{ff}}\left(t\right)$ with $\kappa_{PMMA}$ and G as free parameters, as these are independent of film thickness. The curve fitting is done using least squares nonlinear fitting in Matlab, which is based on the method reported by Coleman and Li [30].

The Si substrates were all cut from the same Si wafer, which has a native oxide layer present. This oxide layer was measured by ellipsometry to be 2.05 nm with 10% measurement error. In Figure 3, which shows a typical sample, this layer is not shown. This means that the reported G includes a contribution from the native oxide. Including the SiO_2_ layer in the thermal modelling offset the result by less than 1%, so this contribution was assumed negligible.

Random and systematic error of the FDTR setup was analyzed. Random error stemming from electrical noise and detectors, lock-in amplifier and vibrations was minimized using ample stabilization times for each sampling frequency, sampling for several seconds at each frequency, and doing three subsequent acquisitions at the same location on each sample. The resulting $\kappa_{eff}$ measured reveals a random error of <1%.

Systematic error can occur due to uncertainties in the values input in the thermal model. These values and their uncertainties were given in Table 1. Some of the values, such as that of the Si substrate, were taken from literature. However, an important exception was made in order to accurately determine the thermal conductivity and volumetric heat capacity of the Au transducer. This is because Malinský et al. showed that gold thin films could be 50% less dense than the bulk value, which severely affects its thermal properties [31]. We determined the properties of the Au transducer film by separate FDTR measurements of reference samples comprising of an 80 nm Au layer deposited on quartz in parallel with the PMMA samples. The thermal properties of the gold film were confirmed by electrical conductivity measurements via the Van der Pauw method [32] and the Wiedemann–Franz Law. The values, given in Table 1, are in good agreement with values reported by Schmidt et al. [16]. The pump and probe laser spot radii were measured with an error of <5%. The assumptions made by the thermal model can also introduce systematic errors. It is assumed that the Si substrate is thermally infinitely thick, so that the signal is not affected by its boundary. This is a fair assumption, since the thermal penetration depth given by $\delta =\surd \frac{D}{\pi f}$ where *D* is thermal diffusivity and *f* is modulation frequency is δ < 50 μm for the Si substrate, which is 500 μm thick. It is also assumed that the pump and probe beams are Gaussian shaped, which is a fair assumption. In the knife’s edge measurement, the experimental data was fit with <3% fit error to a so-called error function generated when a Gaussian beam is blocked by sharp edge in a semi-infinite region [22]. The laser beams are assumed to be well co-aligned, which was done using careful two-step alignment using two 1 mm pinholes separated by a 40 cm distance. The above-mentioned uncertainties resulted in a total experimental error of <10% for G.

## 3. Results and discussion

**Figure 4:**
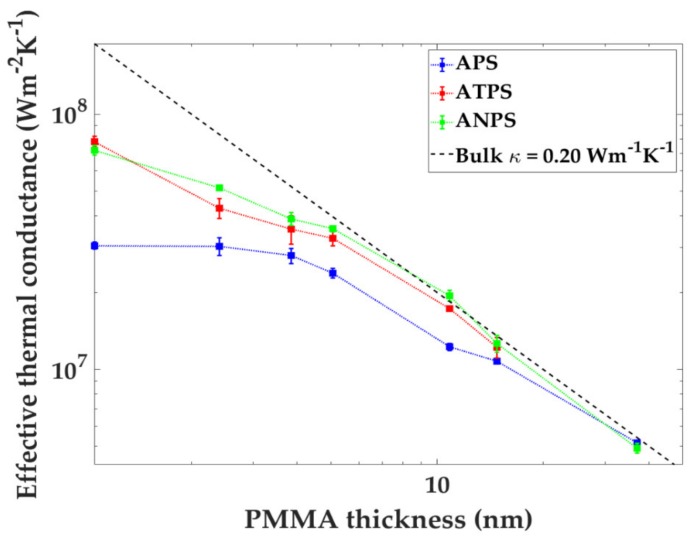
The effective thermal conductance $G_{eff}$ of the PMMA layer as a function of PMMA thickness for APS, Au/Ni/PMMA/Si (ANPS) and Au/Ti/PMMA/Si (ATPS) samples. The dotted lines were added for clarity. Also shown here is the linear relation for bulk PMMA (dashed line). The error bars were calculated by the standard deviation error in the thermal measurements.

In Figure 4, the measured effective thermal conductance ($G_{eff}$ *= 1/*$R_{eff}$) is plotted as a function of PMMA thickness. The equation for the conductance of bulk PMMA, Gefft=κt where κ= 0.20 Wm^−1^·K^−1^, is also shown. Seeing the experimental data points fall below the linear curve below 10 nm demonstrates the importance of using ultra-thin polymer layers in order to see the interface contribution. From the figure it is apparent that when the PMMA thickness is reduced, the effective thermal conductance is lowered. This means that the TBC becomes increasingly dominating relative to the bulk PMMA thermal conductivity. One can imagine a scenario in which the experimental data of a thermal measurement follows the bulk linear curve all the way to *t* = 0. In this scenario, the TBC would be infinite. On the other hand, if the TBC was zero, the $G_{eff}$ would be zero for all *t*. When the APS, ATPS and ANPS are shown together, it gives the reader a visualization of the difference in TBC between the data sets. For APS data, the data falls well below the bulk linear curve. This means that the TBC for APS samples is lower than for the ATPS and ANPS samples.

**Figure 5:**
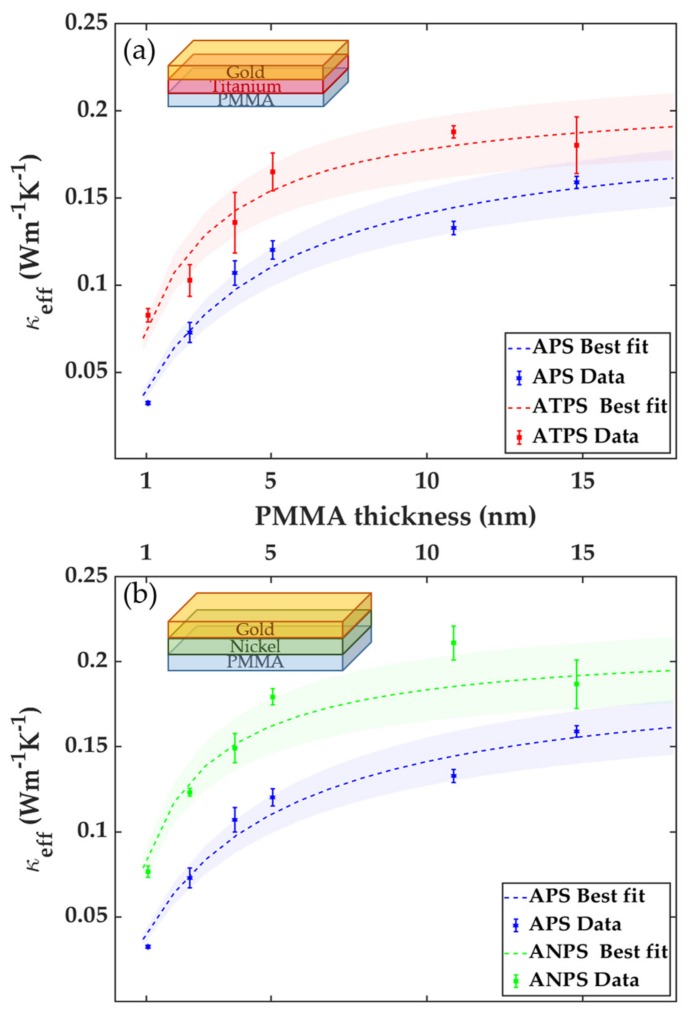
(**a**) Effective thermal conductivity of APS and ATPS samples. (**b**) Effective thermal conductivity of APS and ANPS samples. Dashed lines are the best fit of the experimental data to the series thermal resistance model using $\kappa_{PMMA}$ and G as free parameters. Error bars are calculated from the standard deviation error in the thermal measurements. The shaded area around the fit curve corresponds to the 10% total experimental uncertainty.

The data in Figure 5 was fitted using Equation (2) with $\kappa_{PMMA}$ and G as free parameters. The best fit for $\kappa_{PMMA}$ and G for the three different systems are summarized in the Table 2. The best fit $\kappa_{PMMA}$ values are respectively 1.2% (APS), 5.3% (ATPS) and 5.8% (ANPS) off from the accepted bulk value of thermal conductivity for PMMA (0.20 W·m^−1^·K^−1^) [33].

The G values are within the same order of magnitude as experimental and theoretical values reported by others [8,20,33,34]. The ATPS sample sees a near doubling in G. The increase in G is in agreement with recent work where TiO*_x_* with near-zero oxygen content acted as thermal management at Au/non-metal interfaces [35]. Duda et al. reported that the thermal boundary conductance at Au/Si interface was enhanced by a factor of four by inclusion of a Ti adhesion layer and removal of the native oxide [10].

It is well established that TBC depends on the bonding strength at the interface, which has been predicted by analytical models [36], molecular dynamics simulations [37,38,39,40,41] and experiments [42,43,44,45]. In this study, the observed increase in G is most likely caused by an increase in adhesion between the metal and polymer. Au has poor adhesion to most substrates, including PMMA [9]. This is due to the low reactivity of noble metal Au combined with the low-wetting PMMA surface. This is especially true for spin-coated PMMA, which deposits the polymer chains in a randomly oriented manner. The bonding between Au and as-deposited PMMA is mainly weak van der Waals forces caused by the physical adsorption of Au atoms on the PMMA surface during deposition. However, the PMMA molecule is polar, and the carbonyl (C=O) and methoxy (O–CH_3_) groups have the potential of bonding to metallic atoms through a Lewis acid–base interaction in which the metal acts as a weak base [46]. Ti is commonly used as an adhesion-promoting layer between noble metals such as Au and the substrate [47,48] since it is a more reactive metal that acts as a weak Lewis base that can donate an electron pair. The same holds for Ni, which is also frequently used as an adhesive layer between Au and substrates [49]. Chang et al. reported Ti film as having the highest adhesion strength to all the polymers they studied, with a peel strength at least four times higher than Au, which had the lowest adhesion to the polymers [50]. The appearance of TiC peaks in the X-ray photoelectron spectroscopy (XPS) spectra indicated covalent bonds between Ti atoms and C atoms in PMMA. XPS investigations by Bébin and Prud’homme of Ni nanofilms on PMMA showed that Ni reacted with the oxygen in the PMMA molecule [51]. This led to formation of nickel oxide, and a covalent bond at the metal–polymer interface. Furthermore, Konstadinidis et al. did an XPS study to investigate interactions between evaporated Ti thin films and functional groups at the surface of self-assembled monolayers (SAMs). They found that Ti reacted to form Ti–O–bonds with ester-terminated SAMs and Ti–C–bonds with all the SAMs they studied [52].

In the as-deposited Au/Ti or Au/Ni interface, the phenomenon of thin film diffusion results in an intermetallic compound at the interface, providing an intimate contact between the two metals [53,54]. Metal-metal TBC values reported by Monachon et al. are 1–2 orders of magnitude larger than the TBC we are probing in this work [55]. Thus, in this work we would not expect the additional Ti or Ni layer to increase the measured thermal conductance of the sample due to Au/Ni or Au/Ti TBC. Moreover, the thermal capacity of the Ti/Ni layer was not expected to contribute significantly to the overall thermal conductance, because the layer is only 2 nm thick. Indeed, this was confirmed by varying the volumetric thermal capacity of Ti and Ni reported in Table 1 and observing that the fit has very low sensitivity to this parameter.

Several studies indicate a large potential for increased Au–PMMA bonding and hence improved TBC through post-treatment of the as-deposited PMMA. Common methods are functionalizing the PMMA surface by plasma treatment [56], UV ozone surface modification [27], organic or halogenated solvents [57,58] and silane bridging [59]. The idea is to treat the PMMA such that chemical bonds can form at the Au–polymer interface. In the example of silane, it has the ability to react chemically with both the Au and the PMMA chain through a silicon–methoxy covalent bond.

The Ti adhesion layer is known to increase electron-phonon coupling, an effect that was studied by Giri et al. in a TDTR study of Au–Ti layers on different dielectric substrates [25]. The study found an improved metal-dielectric thermal transfer when adding a 3 nm Ti interface layer. They found that the addition increased the magnitude of the electron-phonon coupling by a factor of almost five. The enhanced electron-phonon coupling only had an effect on the TBC in highly non-equilibrium conditions, when the electron temperature *T_e_* is much higher than the phonon temperature *T_p_*. In the present study, the time scale is such that, *T_e_* ≈ *T_p_*, so the effect of increased electron-phonon coupling is assumed negligible. At near-equilibrium conditions, the study attributed the increased TBC to the improved adhesion due to inclusion of the Ti layer, and also possibly due to an improved overlap in low frequency phonon modes between Ti and the substrates.

In conclusion, we have shown the use of the FDTR technique to measure the thermal properties of a PMMA layer coated with Au. The fit value of bulk thermal conductivity of PMMA is in good agreement with the widely accepted value of 0.20 W·m^−1^·K^−1^ and we have demonstrated experimentally that adding a 2 nm Ti or Ni layer at the Au/PMMA interface can significantly enhance the TBC. Indeed, the TBC increases by a factor of two from 59 × 10^6^ W/m^2^·K (APS) to 115 × 10^6^ W/m^2^·K (ATPS) and 139 × 10^6^ W/m^2^·K (ANPS). This is to our knowledge the first demonstration of tuning the TBC at the metal–polymer interface, and could be useful in thermal management of low-dimensional organic electronics.

## Figures and Tables

**Table 1:** Thermal conductivity κ and volumetric thermal capacity CV values for the Au film were found by measuring reference samples with Au directly deposited on quartz using a two-parameter fit. We measured metal film thicknesses by atomic force microscopy (AFM) on the reference samples. We measured polymethyl methacrylate (PMMA) thicknesses by ellipsometry on Si reference samples with <10% error. The effective thermal conductivity κ of the PMMA layer is the unknown parameter, and hence is not listed in the table.

	Au	Ti	Ni	PMMA	Si
κ (W·m^−1^·K^−1^)	227.80 ± 23 *	8.20 ^a^	52 ^d^	-	148 ^c^
*C_V_* (10^6^ J/m^3^·K)	1.94 ± 0.2 *	3.01 ^a^	3.92 ^e^	1.73 ^b^	1.68 ^c^
*t* (nm)	84.3 ± 3 *	2.4 ± 0.1 *	2.0 ± 0.1 *	1–15	∞

* measured value, ^a^ [25], ^b^ [26], ^c^ [20], ^d^ [27], ^e^ [28,29].

**Table 2:** Best fit values for $\kappa_{PMMA}$ and G for the three systems APS, ANPS and ATPS are summarized below. The values reported are subject to a 10% total experimental error.

System	$\kappa_{PMMA}\;(W\cdot m^{-1}\cdot K^{-1})$	$G\;(W\cdot m^{-2}\cdot K^{-1})$
APS	0.198	59
ATPS	0.211	115
ANPS	0.212	139
